# The Mississippi Valley Dental Association

**Published:** 1894-04

**Authors:** 


### The Mississippi Valley Dental Association.

The forty-ninth annual meeting of the Mississippi Valley Association of Dental Surgeons convened in the lecture room of the Ohio Dental College, pursuant to the following circular sent to the members of the Association:

CINCINNATI, O., March 6, 1894.

*Dear Doctor*:—For several years it has been a question among the members of the Mississippi Valley Association of Dental Surgeons. "Has the society outlived its usefulness?" There was a time when this society accomplished the greatest amount of good, but conditions have changed. City, District, State, National and International organizations have occupied the field. A society representing the Mississippi Valley does not seem a necessity. Men of talent have all their spare time taken up attending to the other organizations mentioned. Last year there was no meeting on account of the Columbian Dental Congress. This year there is, as yet, very little prospect of a successful meeting. The officers do not feel that they care to take the responsibility upon themselves of saying that the society shall or shall not be abandoned. Therefore, let this be notice to all members of the Mississippi Valley Dental Society, that there will be a meeting of this society, for the purpose of deciding upon the continuance or abandonment of the Association.

To be held in the Ohio Dental College Building on the 28th day of March, at 2 P. M., 1894.

DR. O. N. HEISE, President,
82 Garfield Place.

DR. J. R. CALLAHAN,

Chairman Ex. Com.

DR. H. T. SMITH, Secretary,

128 Garfield Place.

Pres. Dr. O. N. Heise called the meeting to order, and after reading the above circular the following resolution offered by Dr. J. Taft was adopted unanimously:

"That it be considered the sense of this meeting that the Mississippi Valley Association of Dental Surgeons be continued."

Drs. J. S. Taylor and Betty, Membership Committee.

The following gentlemen were unanimously elected to membership: W. H. Gillette, H. F. Vandervoort, W. B. Fahnestock, and W. S. Locke, all of Cincinnati.

On motion of Dr. Molyneaux, the annual dues for the present year are to be remitted and new members shall pay only the initiation fee.

On motion the Treasurer was authorized to pay all current bills.

On motion of Dr. Callahan, the date for the next meeting shall be the day following the date of the Commencement of the Ohio Dental College, and shall be held in Cincinnati, and it shall be considered the Semi-Centennial Meeting of the Association.

The following members were present: Drs. J. R. Callahan, J. Taft, C. M. Wright, M. H. Fletcher, G. Molyneaux, A. E. Emminger, Jas. Leslie, F. A. Hunter, H. C. Matlack, W. Taft, E. G. Betty, J. I. Taylor, Chas. Welch, H. A. Smith, O. N. Heise, H. T. Smith, W. B. Fahnestock, H. F. Vandervoort W. H. Gillette, W. S. Locke, J. B. Dillon, W. O. Hulick, and A. G. Rose.

The following officers were elected:

President, Dr. J. Taft, of Cincinnati, O.

1st Vice-President, Dr. W. B. Ames, of Chicago, Ill.

2nd Vice-President, Dr. A. E. Emminger, of Columbus, O.

Treasurer, Dr. F. A. Hunter, of Cincinnati, O.

Corresponding Secretary, Dr. H. C. Matlack, of Cincinnati, O.

Recording Secretary, Dr. H. T. Smith, of Cincinnati, O.

Executive Committee—Drs. J. R. Callahan, Cincinnati, O; M. H. Fletcher Cincinnati, O; and L. E. Custer, Dayton, O.

